# Association between interleukin 10 (IL-10) polymorphisms and leishmaniasis progression: a systematic review and meta-analysis

**DOI:** 10.1038/s41598-022-15377-2

**Published:** 2022-07-01

**Authors:** Renata Rocha da Silva, Fernanda de Santana Fontes Vasconcelos, Débora dos Santos Tavares, Priscila Lima dos Santos

**Affiliations:** 1 Health Sciences Postgraduate Program, Federal University of Sergipe, Aracaju, Brazil; 2 Sciences Applied to Health Postgraduate Program, Federal University of Sergipe, Lagarto, Brazil; 3 Department of Health Education, Federal University of Sergipe, Lagarto, Brazil

**Keywords:** Risk factors, Parasitic infection, Interleukins

## Abstract

Interleukin 10 (IL-10) is associated with the progression of leishmaniasis because it inhibits the leishmanicidal action of macrophages and the production of mediators such as IFN-γ and nitric oxide. Studies have shown that specific polymorphisms are associated with the regulatory role of IL-10 and the development of more relevant clinical forms of leishamaniasis. We performed a systematic review and meta-analysis to determine whether single nucleotide polymorphisms (SNPs) of IL-10 influence the progression of leishmaniasis. The selected articles were read in full and only those consistent with the eligibility criteria were included in our study. Seven studies were eligible according to the inclusion criteria and were included in the present systematic review, but only five were subjected to statistical analysis. The pooled odds ratios showed no significant association between the rs1800871 SNP and the progression of leishmaniasis in all genotype models, including the dominant, recessive, homozygote, heterozygote, and allelic models. Regarding the association between rs1800896 SNP and the progression of leishmaniasis, the pooled odds ratios showed no association under all genotype models. Hence, IL-10 SNPs did not show significant association and were not considered a risk factor for the progression of leishmaniasis.

## Introduction

Leishmaniasis is considered a serious public health problem on four continents: the Americas, Asia, and Africa. It has three general forms: cutaneous leishmaniasis (CL), mucocutaneous leishmaniasis (MCL), and visceral leishmaniasis (VL); these are caused by more than twenty species of *Leishmania* and transmitted by the bite of an infected female Phlebotomine sand fly. *Leishmania* are obligate intracellular protozoan parasites that infect mononuclear phagocytes^1,2^.

CL is the most prevalent clinical form. According to the World Health Organization^3^, there are approximately one million cases worldwide, and the most common clinical manifestations comprise the appearance of painless single or multiple lesions in exposed areas, with well-defined borders. The mucocutaneous form has a different clinical manifestation involving marked destruction of mucosal regions, especially in the upper airways, that can provoke respiratory complications. Three to five percent of CL cases progress to the mucocutaneous form^4^.

The most common clinical manifestations of VL, also known as kala-azar, are fever, weight loss, hepatosplenomegaly, and hematological alterations^5^. The disease is fatal in over 95% of cases if not treated, and is considered the most severe form. It is endemic in 13 American countries, with an average of 3470 cases per year and a 7% fatality rate. In 2019, 97% of all cases were in Brazil^6,7^. The diagnosis is usually based on clinical criteria and specific tests, such as serology for the detection of antibodies and parasite isolation through bone marrow or splenic aspirates. In addition, the Kalazar Detect rK39 test is a rapid assay that can qualitatively determine the presence of antibodies against *Leishmania*^8^.

The clinical forms of the leishmaniases are influenced by the host’s immune response. Upon parasitic infection, the innate immune response is triggered to restrain its proliferation. However, the disease progresses when there is suppression of reactive nitrogen and oxygen intermediates (RNI and ROI) within macrophages, responsible for inhibiting parasitic replication^9^. Dayakar et al.^10^ suggest that the Th1/Th2 dichotomy plays a key role in VL. The development of more relevant clinical forms is associated with the predominance of Th2 cells, TGF-β, IL-4, and IL-10, which impair the antiparasitic activity of macrophages.

Interleukin 10 (IL-10) is a regulatory cytokine produced by T cells, B cells, macrophages, dendritic cells (DCs), and epithelial cells^5^. Nylén et al.^11^ reported that IL-10 in VL patient plasma enhances parasite replication in macrophages and that blocking IL-10 reduces parasite growth. Thus, IL-10 is associated with the more severe clinical manifestations because it inhibits the production of other mediators, such as IFN-γ and nitric oxide, and inhibits the leishmanicidal action of macrophages, preventing the elimination of intracellular parasites^5^. On the contrary, there are interleukins associated with controlling leishmaniasis, including IL-12, IL-17, IFN-γ, and TNF-α, that activate the acquired immune response^12,13^.

Single nucleotide polymorphisms (SNPs) are relevant in specific phenotypes that are more prone to develop diseases, considered risk factors^14^. These genetic factors are associated with distinct clinical manifestations of several diseases, and they generate important phenotypic differences in the population by coding proteins in different amounts or causing modification of the proteins themselves. Thus, the immune response can be induced in different ways depending on the polymorphism involved^15^.

Previous studies have shown that specific polymorphisms are associated with the regulatory role of IL-10 and disease progression^16,17^ several IL-10 polymorphisms have been investigated regarding their roles in leishmaniasis, but the results are inconsistent. Hence, the aim of the present study was to perform a systematic review and meta-analysis to determine if IL-10 SNPs influence the progression of leishmaniasis.

## Materials and methods

This study was conducted following the Meta-analysis Of Observational Studies in Epidemiology (MOOSE) statement^18^ and Preferred Reporting Items for Systematic Reviews and Meta-Analyses (PRISMA) guidelines^19^. This systematic review was registered in the PROSPERO database (CRD42021255374).

### Research question and eligibility criteria

The present review focused on the subsequent question: Are the IL-10 polymorphisms risk factors for the progression of leishmaniasis? To evaluate the association between these variables, we assumed that all subjects were in an endemic area and allocated in groups (case or control) according to the stage of infection. The case group was composed of *Leishmania*-positive subjects with clinical manifestations of the disease, and the control group included asymptomatic individuals (*Leishmania*-positive) for VL and healthy individuals (*Leishmania*-negative) for CL. Studies were considered eligible if they satisfied the subsequent criteria: (i) case–control studies; (ii) subjects diagnosed with leishmaniasis by Montenegro skin test, myelogram, polymerase chain reaction (PCR), or serology tests, regardless of age and gender; (iii) SNP polymorphisms were investigated factors; (iv) control group composed of asymptomatic subjects as described above; and (v) sufficient information to evaluate the association between IL-10 polymorphism expression and disease progression. Family-based studies, editorials, comments and opinions, articles of reflection, projects, technical reports, reviews, and articles not related to IL-10 polymorphisms and leishmaniasis were excluded from this review.

### Data search strategy

A systematic search using ScienceDirect, PubMed, and Scopus databases was performed to identify all relevant publications regarding the association of IL-10 SNPs in all types of leishmaniasis up to April 2021. The structured search strategy used the following terms: (“Interleukin 10” OR “IL-10”) AND (“polymorphism” OR “gene” OR “variant”) AND (“leishmaniasis” OR “visceral leishmaniasis” OR “cutaneous leishmaniasis” OR “mucocutaneous leishmaniasis). To expand the number of eligible articles retrieved, no restrictions were imposed regarding study design or language.

### Study selection

Two reviewers (R.R.S. and F.S.F.V.) independently screened the search results and identified studies that were potentially relevant based on the paper’s title and abstract. The selected articles were read in full and only the relevant ones, consistent with the eligibility criteria, were included in our study. Any disagreements were resolved after discussion with a third reviewer (D.S.T.). The reference lists of the retrieved studies were manually scanned to identify additional relevant studies.

### Data extraction

Data extraction from each manuscript was performed in consistence with the eligibility criteria, and the extracted data was recorded in a spreadsheet. The following were recorded from each article: name of the author(s), year of publication, total number of subjects (cases/controls), country where the research was conducted, leishmaniasis form, and IL-10 polymorphisms. We also listed genotype and allele counts for cases/controls and p values.

### Risk of bias assessment

Studies that met the eligibility criteria were assessed for methodological quality using the Newcastle–Ottawa Scale (NOS)^20^ by two independent reviewers (R.R.S. and F.S.F.V.). The NOS scale is made through a “star system” and judges the study based on three perspectives: selection of the study groups (maximum of one star for each column), the comparability of the groups (maximum of two stars), and the exposure (maximum of one star for each column). Studies that received a score of 6 or higher were considered as high quality.

### Statistical analysis

For articles that studied more than one polymorphism with different control groups, each result was considered individually for meta-analysis. Odds ratios (ORs) were used in meta-analysis to test the association between each SNP and the progression of leishmaniasis in case–control studies; 95% confidence intervals were included and p values < 0.05 were considered statistically significant. Five genetic models for each SNP were investigated corresponding to dominant, recessive, homozygote, heterozygote, and allelic modes of inheritance. Heterogeneity was evaluated using the chi-square-based Cochran’s Q statistic and the I^2^ index^21^. When high heterogeneity (p < 0.1 for the Q test or I^2^ > 50%) was detected, the random-effects model was employed for meta-analysis; otherwise, a fixed-effects model was applied^22^.

Sensitivity analysis was performed by excluding individual studies from the pooled ORs and recalculating the statistical significance to explicate the stability of findings and to ascertain whether final pooled effect sizes were affected by a single publication. Since the number of included studies was less than 10^23^, publication bias was estimated by the qualitative effect of funding sources. Pearson’s chi-squared test was applied to examine Hardy–Weinberg equilibrium (HWE) in the healthy control group. The HWE was reached if p > 0.05. In all analyses, Review Manager Version 5.3 software (Cochrane Collaboration, Copenhagen) was used.

## Results

### Study selection

**Figure 1:**
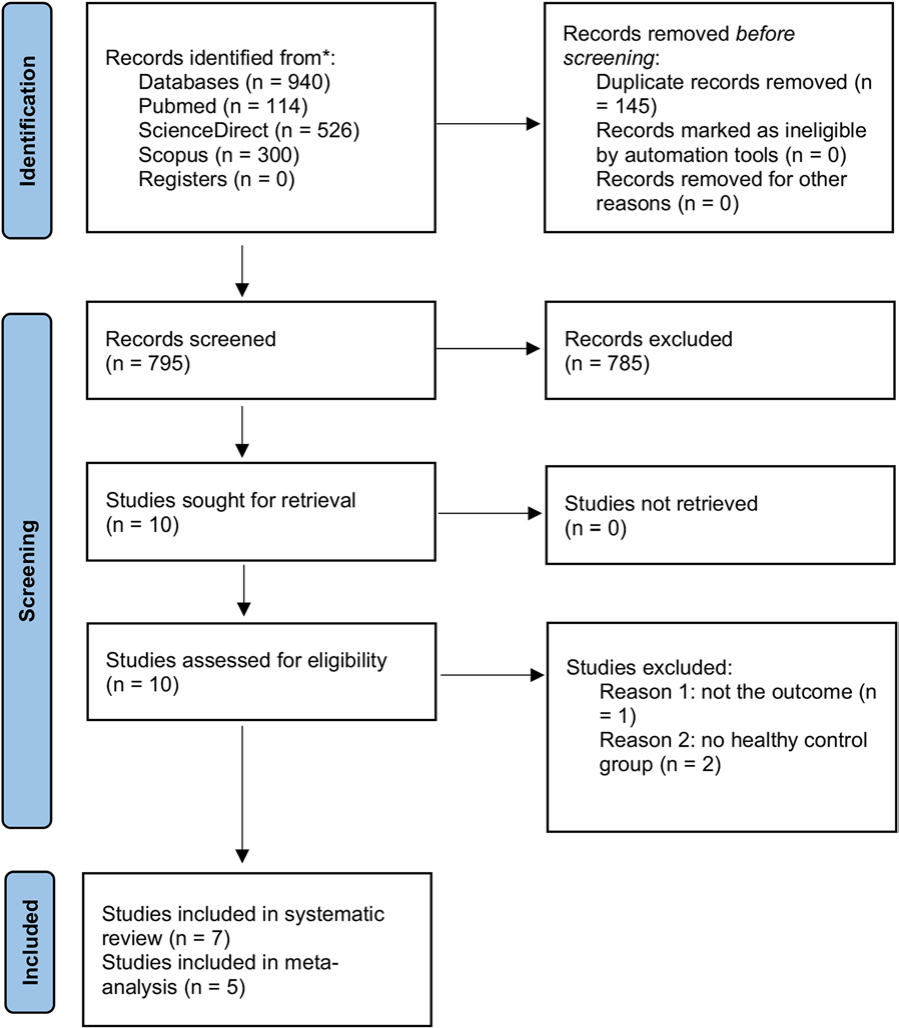
Flowchart of the literature search and screening process.

The initial search yielded 940 articles; 526 were collected from ScienceDirect, 114 from PubMed, and 300 from Scopus. After removing the duplicates, 795 publications remained. Of these, 787 were excluded either by title and abstracts (n = 785) or full text (n = 2) evaluations. Finally, seven studies^24–30^ were eligible according to the inclusion criteria and were included in this systematic review, but only five studies were subjected to statistical analysis (Fig. 1). The studies excluded from the meta-analysis had polymorphisms which the other included studies did not cover; therefore, it was not possible to compare them^28^. The references of all eligible publications were crosschecked, and no further records were found to meet the inclusion criteria.

All eligible studies were published between 2013 and 2020. Two of the included studies were conducted in Iran^25,30^, three in Brazil^24,26,27^, one in India^29^, and one in Turkey^28^. Table 1 summarizes the characteristics of the studies included in the systematic review, and Table 2 shows the genotype and allele frequencies reported in the studies included in the meta-analysis.

**Table 1:** Characteristics of studies included in a meta-analysis for the association of IL-10 SNPs and leishmaniasis.

Study	Year	Country	Form of leishmaniasis	Cases	Control	Polymorphisms studied	Funding source
Covas	2013	Brazil	Cutaneous	110	682	rs1800871	Public
Hajilooi	2013	Iran	Visceral	100	190	rs1800871	Public
Hajilooi	2014	Iran	Visceral	110	187	rs1800896	Public
Kirik	2020	Turkey	Cutaneous	55	110	rs1800896	Public
Mishra	2015	India	Visceral	184	172	rs1518111	Public
						rs1554286	
						rs3024496	
						rs3024498	
Shehadeh	2017	Brazil	Cutaneous	102	150	rs1800871	Public
						rs1800872	
						rs1800896	
Sonon	2019	Brazil	Cutaneous	114	346	rs1800871	Public
						− 657

**Table 2:** Distribution of genotype and allele from studies included in the meta-analysis among leishmaniasis patients and controls.

Study author	Cases					Control					HWE p-value
	TT	TC	CC	T	C	TT	TC	CC	T	C	
***rs1800871***											
Hajilooi et al.^25^	2	96	2	100	100	28	140	22	184	196	< 0.0001
Covas et al.^26^	12	54	40	78	134	86	248	237	420	722	0.11
Shehadeh et al.^27^	10	42	50	62	142	17	60	73	94	206	0.38

Study author	Cases					Control					HWE p-value
	AA	AG	GG	A	G	AA	AG	GG	A	G	
***rs1800896***											
Hajilooi et al.^30^	0	102	8	102	118	10	142	35	162	212	< 0.0001
Shehadeh et al.^27^	46	38	18	130	74	62	67	21	191	109	0.67
Kirik et al.^28^	6	49	0	61	49	5	104	1	104	106	< 0.0001

Sonon et al.^24^ and Covas et al.^26^, both analysing the rs180071 IL-10 SNP, indicated that IL-10 is not associated with CL and that the SNP does not regulate IL-10 secretion. Kirik et al.^28^ analysed the rs1800896 SNP and presented a deviation from HWE both in patient and control groups, and thus could not compare the risk estimate results. Both Hajilooi et al.^25,30^ studies demonstrated SNP influence in the progression of VL, particularly, rs1800871 and 1800896 IL-10 SNPs, respectively. Mishra et al.^29^ demonstrated that of the four SNPs analysed, only rs3024498 showed association with VL. However, Shehadeh et al.^27^ presented no association of IL-10 polymorphisms and VL progression, neither with rs1800871 nor rs1800896.

### Quality of the studies

All studies included in this systematic review had an overall good methodological quality with NOS scores ranging from 6 to 8. All studies (100%) stated a research question with adequate case and control definitions, and clearly described information related to the eligibility criteria with the same method as for assessing the SNPs between the groups. A deficiency in the representativeness of the cases was seen, with 38% of the studies being without a star (Table 3).

**Table 3:** Risk of bias of the included studies in the systematic review using the tool Newcastle–Ottawa (NOS) quality assessment scale for case–control studies.

Study	Selection				Comparability	Exposure		
	1	2	3	4	1	1	2	3
Covas	✩	–	✩	✩	✩	✩	✩	–
Hajilooi	✩	–	✩	✩	✩✩	✩	✩	–
Hajilooi	✩	✩	✩	✩	✩✩	✩	✩	–
Mishra	✩	✩	✩	✩	✩✩	✩	✩	–
Shehadeh	✩	✩	✩	✩	–	✩	✩	–
Sonon	✩	✩	–	✩	✩✩	✩	✩	–
Kirik	✩	✩	✩	✩	✩✩	✩	✩	–

Selection: 1. Adequate case definition; 2. Representativeness of the cases; 3. Selection of controls; 4. Definition of controls. Comparability: 1. Comparability of cases and controls on the basis of the design or analysis. Exposure: 1. Ascertainment of exposure; 2. The same method of ascertainment for cases and controls; 3; Non-response rate.

### rs1800871 polymorphism

**Figure 2:**
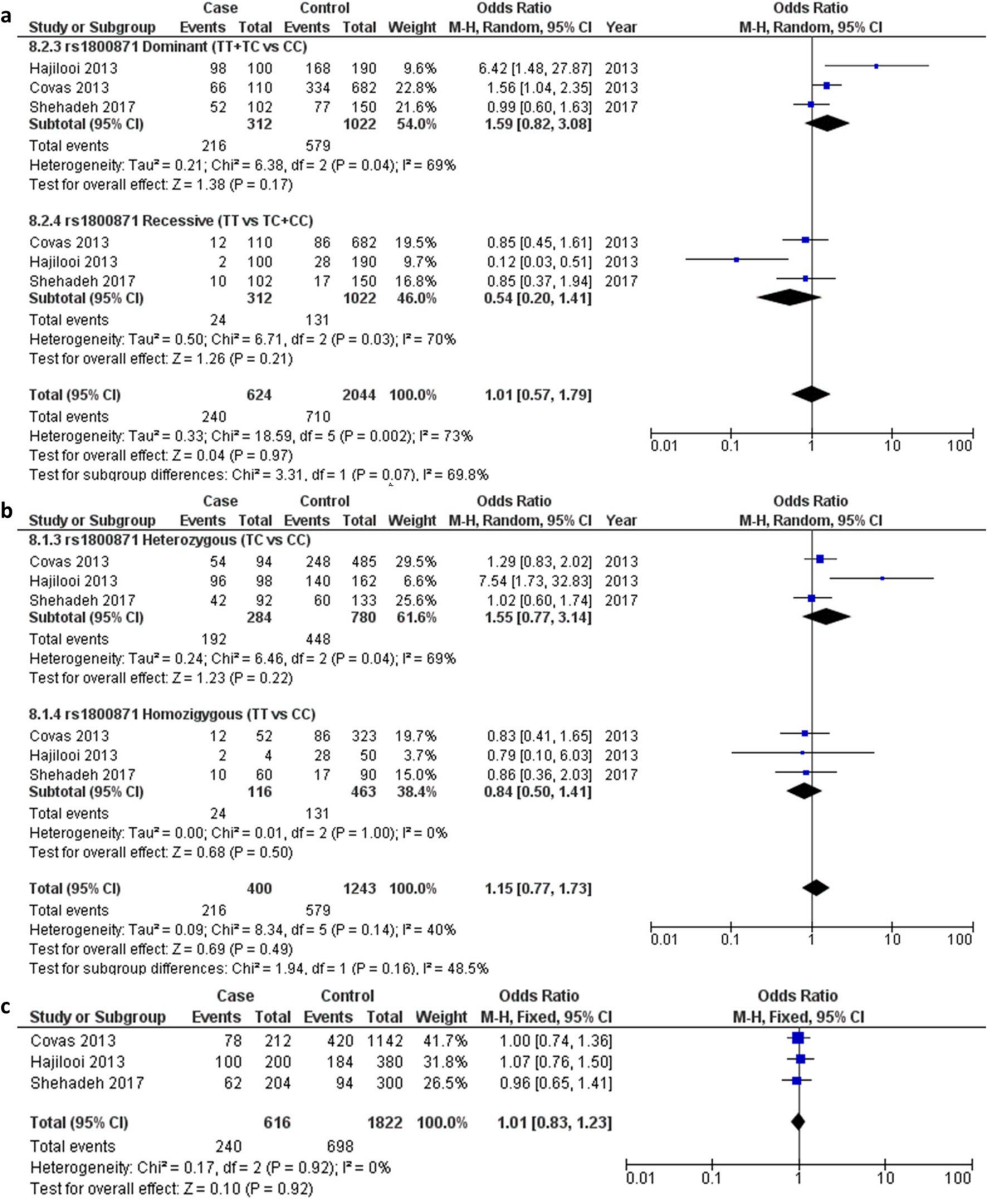
Pooled odds ratios and 95% confidence intervals for the association between the rs1800871 polymorphism and the progression of leishmaniasis. (**a**) Dominant and recessive models. (**b**) Homozygous and heterozygous models. (**c**) Allelic model.

Overall, three studies containing 312 cases and 1022 healthy controls were found to be eligible and included for quantitative analysis of association between the rs1800871 SNP and leishmaniasis progression^24–27^. Among these, one study analysed the VL form and three studies the CL form; these were included in the subgroup analysis. The pooled ORs showed no significant association between the rs1800871 SNP and leishmaniasis progression across all genotype models, including the dominant (OR 1.59, 95% CI 0.82–3.08, I^2^ = 69%, *p* = 0.04) and recessive models (OR 0.54, 95% CI 0.20–1.41, I^2^ = 70%, *p* = 0.03) (Fig. 2a), homozygote (OR 0.8, 95% CI 0.50–1.41, I^2^ = 0%, *p* = 1.00) and heterozygote models (OR 1.55, 95% CI 0.77–3.14, I^2^ = 69%, *p* = 0.04) (Fig. 2b), and allelic model (OR 1.01, 95% CI 0.83–1.23, I^2^ = 0%, *p* = 0.92) (Fig. 2c). Although the control genotype in one study was not within HWE, the significance of the association outcome was maintained when this study was omitted from the meta-analysis.

### rs1800896 polymorphism

**Figure 3:**
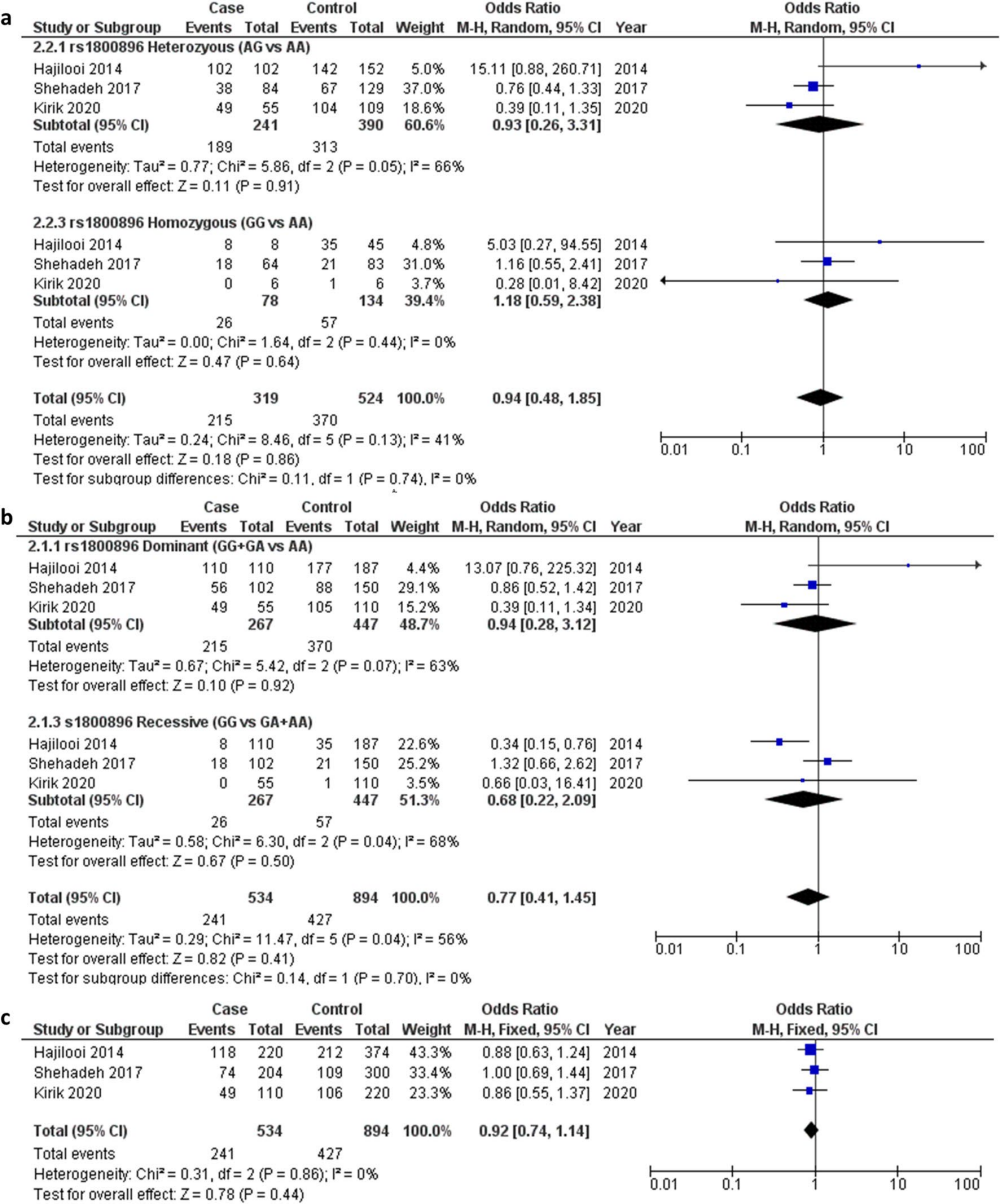
Pooled odds ratios and 95% confidence intervals for the association between the rs1800896 polymorphism and the progression of leishmaniasis. (**a**) Homozygous and heterozygous models (**b**) Dominant and recessive models. (**c**) Allelic model.

Herein, three studies involving 267 cases and 447 controls were included in the final analysis of association between the rs1800896 SNP and leishmaniasis progression^28,29,31^. Among these studies, one analysed the VL form and two the CL form. The pooled ORs did not show association under all genotype models, including the dominant (OR 0.94, 95% CI 0.28–3.12, I^2^ = 63%, *P* = 0.07) and recessive models (OR 0.68, 95% CI 0.22–2.09, I^2^ = 68%, *p* = 0.04) (Fig. 3a), homozygote (OR 1.18, 95% CI 0.59–2.38, I^2^ = 0%, *p* = 0.44) and heterozygote models (OR 0.93, 95% CI 0.26–3.31, I^2^ = 66%, *p* = 0.05) (Fig. 3b), and allelic model (OR 0.92, 95% CI 0.74–1.14, I^2^ = 0%, *p* = 0.86) (Fig. 3c).

The controls of two studies included in this conventional meta-analysis deviated from HWE but did not significantly influence the overall association result (Table 2).

### Sensitivity analysis and publication bias

Sensitivity analysis was performed to assess the influence of each individual study on the pooled ORs by sequential omission of individual studies. However, the corresponding pooled ORs were not materially altered on removal of any individual study. Moreover, in all tests, the value for heterogeneity was not reduced. Therefore, the sensitivity analysis confirmed that the results of this meta-analysis were statistically reliable and stable. There was no potential for publication bias, considering that all studies had public sources of funding (Table 1).

## Discussion

This is the first meta-analysis of the association between three IL-10 SNPs and leishmania progression. Alavarado-Arnez et al.^31^ showed association between IL-10 SNPs -819 C/T allele and -592 C/A allele and risk of leprosy. Ni et al.^32^ presented an association between IL-10 1082 G allele and risk of developing gastric cancer. Although other meta-analyses have shown the association between IL-10 polymorphisms and other diseases^16,31–35^, the present meta-analysis of five case–control studies did not indicate IL-10 SNPs as a risk factor or protective factor for the progression of leishmaniasis.

The clinical and epidemiological complexities of leishmaniasis make it difficult to monitor the disease and distinguish its various stages by identifying biomarkers, since immune responses are relative. High levels of IL-10 are associated with disease progression and high parasite load, and these levels are reduced with successful treatment. Studies have revealed the association between this biomarker and the suppression of the immune response; IL-10 acts in the inhibition of antigen presentation, inactivating macrophages and, consequently, making it impossible to eliminate the parasite^36,37^.

The development of leishmaniasis is related to the immune profile of the individual. The severity and onset of clinical manifestations depend on responses that are not yet fully understood; however, the importance of the action of cytokines in the control and progression of the disease is known. Polymorphisms in the genes of interleukins related to the immune system may be responsible for disease progression; since the C/C genotype is associated with higher IL-10 production, the C allele may be related to the development of leishmaniasis^38^. Salhi et al.^39^ demonstrated that the − rs1800871 C/C polymorphism located in the IL-10 promoter region may be related to the increased expression of this cytokine and, consequently, associated with a higher risk of lesions in American Tegumentary Leishmaniasis patients.

Studies in Iran have shown that there is an association between the IL-10 SNP at position − 819 (rs1800871) and the deficit in immune response leading to the chronicity and severe manifestations of VL. This was more evident in heterozygous C/T patients than in homozygous patients, but there is not enough data to explain this difference^24,29^.

This meta-analysis found that the SNP at the − 819 position did not show a relationship with poor prognosis in any genotype model, whether dominant, recessive, allelic, homozygous, or heterozygous, which supports other studies that have evaluated CL and concluded that the allele frequencies did not show remarkable differences between patients and controls^22,26^.

Regarding the rs1800896 polymorphism studied, there was also no significant difference between patients and controls. Although the research has been carried out in several locations, since vulnerability to CL may not be related only to this SNP, but to several other aspects, such as ethnicity, species of *Leishmania*, and geographic territory. This study did not show a relationship with any model and may emphasize that this polymorphism (rs1800896) cannot explain all forms of immune responses control in the patient. Innate and acquired immunity and other cytokines may also be involved in the pathogenesis^27,30^.

Finally, it is important to consider the limitations of this study when analysing the results. There are few studies published on IL-10 polymorphisms and leishmaniasis, and most of them did not report any standardization on control groups. Moreover, small sample sizes and different methodological strategies, such as sample collection, ethnicity of subjects, forms of leishmaniasis, and sample processing of the included studies, may have affected the data analyses and the final heterogeneity results in some forests plots. Publication bias, which occurs when the authors support the interests of the private study’s financial sponsor, along with conflicts of interest, which may affect study design, conducting, and reporting (contributing to the omission of results or underreporting negative results^23^, was investigated in a qualitative form, due to the insufficient number of primary studies in the literature. However, this is most likely not an issue herein since all studies had public funding and authors did not report conflicts of interest.

Despite the evidence of the role of IL-10 in the persistence of leishmaniasis regardless of immune response, our findings demonstrate no significant association between rs1800871 and rs1800896 and the progression of leishmaniasis. Nevertheless, future research on the subject with greater methodological rigor and sample sizes is mandatory to clarify the role of IL-10 and its genetic profiles on disease progression and treatment^26,29^.
